# Myrrhone exert cardioprotective effect against isoproterenol-induced myocardial injury in rats via alteration of NF-κB and Bax/Bcl-2/caspase-3 signaling

**DOI:** 10.1590/acb408225

**Published:** 2025-11-17

**Authors:** Lin Lin, Jinhua Shao

**Affiliations:** 1 Xi’an No. 5 Hospital – Department of Cardiovascular – Xi’an – China.

**Keywords:** Myocardial Reperfusion Injury, Inflammation, Matrix Metalloproteinases, Apoptosis, Oxidative Stress

## Abstract

**Purpose:**

To examine the cardioprotective effects of myrrhone against isoproterenol (ISO)-induced myocardial injury in rats.

**Methods:**

Myocardial injury was induced in the rats through subcutaneous administration of ISO (85 mg/kg). The body weight, heart weight, electrocardiogram (ECG), cardiac, antioxidant, electrolyte, membrane-bound enzymes, antioxidant, cytokines, and inflammatory parameters were estimated. The mRNA expression of inflammatory parameters was estimated in the cardiac tissue.

**Results:**

Myrrhone treatment significantly (*p* < 0.001) altered ECG parameters, body weight, heart weight, and heart weight/body weight ratio. Myrrhone significantly (*p* < 0.001) improved the level of 5-hydroxytryptamine (5-HT) and suppressed the level of cardiac parameters like creatine kinase-MB, creatine kinase, lactate dehydrogenase, cardiac troponin I, and cardiac troponin T. It also suppressed the level of hepatic parameters such as aspartate aminotransferase, and alanine transaminase; altered the electrolyte, membrane-bound enzymes, and antioxidant parameters. Myrrhone treatment significantly (*p* < 0.001) altered the level of cytokines such as tumor necrosis factor-α (TNF-α), interleukin (IL)-10, IL-1β, IL-17, and IL-6; inflammatory parameters like cyclooxygenase-2, prostaglandin, inducible nitric oxide synthetase, nuclear factor kappa-light-chain enhancer of activated B cells; matrix metalloproteinases such as 2, and 9; apoptosis parameters viz., Bcl-2-associated X protein (Bax), B-cell lymphoma 2 protein (Bcl-2), and caspase-3 parameters. Myrrhone treatment significantly (*p* < 0.001) altered the mRNA expression IL-6, IL-1β, IL-10, TNF-α, Bcl-2, caspase-3, Bax, and caspase-9.

**Conclusion:**

Myrrhone exhibited the cardioprotective effect against ISO-induced myocardial injury in rats via alteration of kappa-light-chain enhancer of activated B cells (NF-κB) and Bax/Bcl-2/caspase-3 signaling.

## Introduction

It is widely known that the heart is a vital organ, and any dysfunction in its tissue can have significantly effects on other organs^1^. Myocardial injury is a major cause of morbidity and mortality in both developing and developed countries^2^,^3^. Myocardial injury, commonly known as a heart attack, is a serious medical condition that occurs when the blood supply to portion of the heart tissue is blocked. This obstruction prevents oxygen and nutrients from reaching the heart muscle, resulting in tissue damage or death^4^. Plaque accumulation in the coronary arteries is the source of this occlusion, which can result in either total or partial arterial obstruction^5^. There is a mismatch between the demands of the heart for oxygenated blood and the ability of the coronary arteries to meet that demand resulting from coronary artery blockage, which is the root cause of myocardial injury^6^. The heart has limited capacity for anaerobic metabolism and cannot compensate the lack of oxygen, blood, and the necessary nutrients during a myocardial damage. This inadequacy tends to produce a pathologic change followed by congestive heart failure^4^.

However, myocardial injury condition can induce the various symptoms such as myocardial fibrosis and hypertrophy. Previous reports suggest that apoptosis, oxidative stress, and inflammatory reactions are involved in the mechanism of myocardial injury^7^. Previous research indicates that isoproterenol (ISO)-induced oxidative stress is mediated by free radicals or reactive oxygen species (ROS). This is evidenced by a significant increase in tissue lipid peroxidation (LPO) and a reduction in antioxidant parameters. Antioxidants play a crucial role in protecting against myocardial injury. It is believed that any phytoconstituent or compound with potent antioxidant activity can safeguard cardiac tissue from myocardial injury^7^,^8^.

During the myocardial injury, increased production of cytokines leads to tissue infiltration by inflammatory cells. Inflammatory cytokines can accelerate myocardial injury, often occurring alongside severe congestive heart diseases^9^. During the myocardial injury, neutrophils infiltrate the infarcted area, exacerbating injury to myocardial cells by triggering the production of cytokines, proteolytic enzymes, and chemokines^10^. Ultimately, this cascade leads to increased ROS generation and heightened oxidative stress. Elevated levels of cytokines, ROS, and chemokines can lead to impaired organ function. Cytokines, particularly tumor necrosis factor-α (TNF-α), enhance neutrophil migration into the ischemic area of infarcted myocardial tissue^11^. Interleukin (IL)-6 and IL-1β involved in the inflammatory response to stress in the myocardial tissue. In myocardial injury condition, adverse reactions are classified based on the activation of cellular signaling molecules, including nuclear factor kappa-light-chain enhancer of activated B cells (NF-κB). NF-κB is a well-known key regulator during inflammatory and apoptotic conditions^12^. During myocardial injury, NF-κB phosphorylation triggers an intracellular signaling cascade, leading to the induction of inflammatory cytokines such as TNF-α, IL-1β, and IL-6, along with other inflammation-related proteins. These changes contribute to the pathophysiology associated with myocardial injury^9^.

ISO (1-3,4-dihydroxyphenyl-2-isopropylamino ethanol hydrochloride) is a synthetic catecholamine and β-adrenoceptor agonist, which is commonly used in the treatment of ventricular bradycardia, bronchial asthma, glaucoma, allergic emergencies and finally induces the cardiac arrest^7^,^13^. Therefore, a high dose of ISO induces myocardial stress by depleting the energy stored in cardiomyocytes, ultimately leading to infarct-like necrosis and cellular injury. Following autooxidation, ISO generates a large amount of free radicals, altering the endogenous antioxidant levels in the tissues^7^. The subcutaneous administration of ISO induces the myocardial injury in the rodent. The acute hemodynamic and electrocardiographic changes observed in ISO-induced myocardial injury closely resemble those seen in patients experiencing myocardial injury^4^,^14^. ISO is a well-established model with high validity, reproducibility, and low mortality compared to other rodent models.

The lock-and-key concept describes how allopathic Western drugs work, focusing on a specific metabolic or signaling system. However, the inability to effectively treat diseases often stems from the involvement of multiple molecular mechanisms^13^. Many synthetic drugs have been investigated for their efficacy against myocardial injury, but unfortunately, most of them exhibit side effects, and in some cases, they can even lead to patient’s mortality. These synthetic drugs manifest various undesirable effects that pose serious health risks^15^. Currently, researchers are directing their efforts toward studying plant extracts and their phytoconstituents as potential cardioprotective agents against myocardial injury injuries^16^.

Myrrhone (naphtho2,1-b.furan-6(7H)-one, 8,9-dihydro-1,5,8-trimethyl-, (8R)-) is isolated from *Commiphora myrrha* tree^17^,^18^. It is a common Chinese herbal medicine with various physiological properties, which play a crucial role in the liver damage and renal cancer^19^. Myrrhone exhibited the antioxidant and anti-inflammatory potential against various diseases. In this experimental study, we investigated the cardioprotective effect against ISO-induced myocardial injury in rats and explored the underlying mechanism.

## Methods

### Experimental rats

Swiss Wistar rats (12–14 weeks old, weighing 200 ± 20 g, male) were used in this study. They were maintained under standard laboratory conditions (temperature 22 ± 5°C, relative humidity = 65%, 12/12-h dark/light cycle) and provided with a standard chow pellet diet and water *ad libitum*. The rats were acclimatized to the laboratory environment for seven days prior to the experimental study. The entire study was conducted in accordance with the institution’s Ethical Committee Guidelines.

### Toxicant and tested drug preparation

Subcutaneous administration of ISO (85 mg/kg) was used to induce the myocardial injury^20^. The tested drug (myrrhone) was administered to the rats as an oral suspension, prepared by dissolving the drug in 1% carboxymethyl cellulose (CMC).

### Experimental group

**Figure 1:**
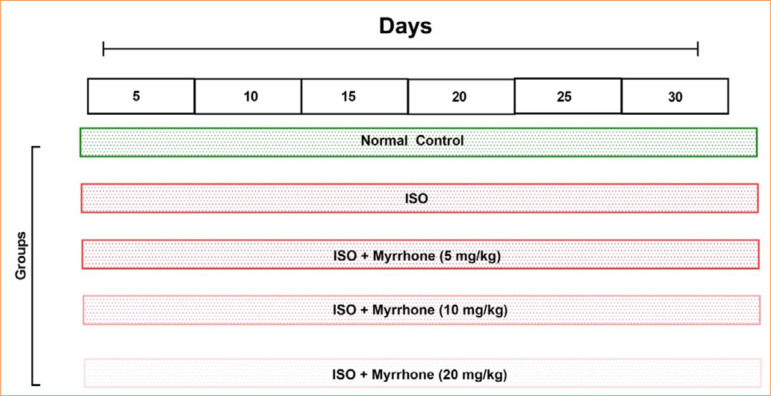
Schematic group of treatment.

The rats were randomly divided into following groups (n = 6). The groups are presented in Fig. 1. The ISO was administered to the experimental rats for two successive days as per the previous reported method with minor modification4,20.

ISO: isoproterenol. Source: Elaborated by the authors.

### Electrocardiography recording

At the end of the experimental study (30 days), the rats were anesthetized with urethane (125 mg/kg, b.w.) and given a stabilization period of 30 minutes before recording electrocardiography^20^. Electrocardiography (ECG) parameters were recorded using an ECG coupler and a student’s physiograph. Briefly, electrodes made from 26-gauge hypodermic needles were attached to both the front and hind paws. One precordial lead was used and placed in a position corresponding to V4 in humans. The QT interval, RR interval, corrected QT interval, QRS interval, and heart rate were estimated.

### Blood and plasma sample preparation

At the end of the study, the rats were anesthetized with ketamine (80 mg/kg) to collect blood samples from the retro-orbital plexus. The blood samples were collected in dry test tubes and allowed to coagulate at room temperature for 30 minutes. After coagulation, the samples were centrifuged for 10 minutes at 5,000 rpm.

### Cardiac parameters

Cardiac parameters such as cardiac troponin T (cTnT) (Cat. No. E-EL-H0503, Elabscience), creatine kinase-MB (CK-MB) (Cat. No. E1343Hu, BT Lab), creatine kinase (CK) (MBS703319, MyBioSource), cardiac troponin I (cTnI) (Cat. No. ab200016, Abcam), and lactate dehydrogenase (LDH) (Cat. No. E-EL-H6004, Elabscience) were estimated using enzyme-linked immunosorbent assay (ELISA) kits, following the manufacturer’s instructions.

### Cardiac 5HT, serum 5HT and cardiac total Ca^2+^

The level of serum and cardiac 5-HT (Cat. No. KLU0048, Krishgen GENLISA) were estimated using the ELISA kits following the manufacture’s instruction.

### Lipid parameters

The level of lipid parameters like triglycerides (TG), total cholesterol (TC), and high-density lipoprotein cholesterol (HDL-C) were estimated using the ELISA kits following the manufacture’s instruction. The level of low-density lipoprotein cholesterol (LDL-C) and very low density lipoprotein cholesterol (VLDL-C) were estimated using the previous formula of Friedewald et al.^21^.

### Hepatic parameters

A previously reported method was used for the estimation of hepatic parameters with minor modifications^7^. Briefly, 100-µL serum sample was mixed into the 100 mM L-aspartate (0.5 mL) and 100 mM α-oxoglutarate in phosphate buffer at pH = 7.4 and left for incubation at room temperature (37°C) for 30 min and mix the 0.5-mL 2,4 dinitrophenylhydrazine and re-incubated at room temperature against serum free reagent (blank sample). The enzyme activity was assessed by employing a standard curve based on commercially available standards, and the results were expressed in international units per liter (IU/L).

### Electrolytes and membrane-stabilizing enzyme

The electrolytes such as potassium (K^+^), sodium (Na^+^), calcium (Ca^2+^), and chlorine (Cl^-^) (Cat. No 2114) were determined using an automated electrolyte analyzer based on the ion selective electrode technology. Briefly, the blood samples were collected in all group rats and allowed to clot and centrifuged for 10 min at 3,000 rpm to collect the serum. The serum samples were then analyzed as the manufacture’s instruction (Roche Diagnostics, United States of America).

The membrane stabilizing enzymes like Mg^2+^ATPase, Na^+^/K^+^ ATPase, and Ca^2+^ATPase were determined by the spectrophotometrically via determining the inorganic phosphate (Pi) liverbated during the ATP hydrolysis using the previous report method with minor modification^22^,^23^. Briefly, we prepared the tissue homogenates in a suitable ice-cold buffer and centrifuged them at 10,000 rpm for 5 min to obtain the microsomal fraction. The reaction mixture contained the appropriate level of Tris HCl buffer, NaCl, MgCl_2_, CaCl_2_, and KCl, depending on the specific enzyme being determined. ATP was added in the reaction mixture to initiate the reaction and was incubated at room temperature (37°C) for defined time. For the termination of the reaction, adding the cold trichloroacetic acid (TCA), the level of Pi generated was quantified. Finally, the enzyme activities were presented as µmol Pi liberated per mg protein per hour.

### Inflammatory cytokines, apoptosis, MMP, and oxidative stress parameters

Inflammatory cytokines such as TNF-α (Cat. No. NBP1-91170, Novus Biologicals), IL-1β (Cat. No. EKF57064-96T, Biomatik), IL-17 (Cat. No. ELH-IL17A-1, RayBiotech), IL-6 (Cat. No. ELH-IL6-1, RayBiotech), and IL-10 (Cat. No. 950.060.192, Medix Biochemica); inflammatory parameters like cyclooxygenase-2 (COX-2) (Cat. No. EH125RB, Thermo Fisher Scientific), prostaglandin (PGE2) (Cat. No. 514010, Cayman Chemical), inducible nitric oxide synthetase (iNOS) (Cat. No. EEL035, Thermo Fisher Scientific), and NF-κB (Cat. No. 85-86081-11, Thermo Fisher Scientific); apoptosis marker viz., B-cell lymphoma 2 protein (Bcl-2) (Cat. No. BMS244-3, Thermo Fisher Scientific), Bax (Cat. No. EEL030, Thermo Fisher Scientific), caspase-3 (Cat. No. KHO1091, Thermo Fisher Scientific); oxidative stress parameters, including catalase (CAT) (Cat. No. A007-1-1, Nanjing Jiancheng Bioengineering Institute, Nanjing), superoxide dismutase (SOD) (Cat. No. 19160, Cayman Chemical), glutathione peroxidase (GPx) (Cat. No. CGP1, Sigma-Aldrich), reduced glutathione (GSH) (Cat. No. CS0260, Sigma-Aldrich), and malondialdehyde (MDA) (Cat. No. MAK085, Sigma-Aldrich); and MMP parameters such as MMP-2 (Cat. No. MMP200, Thermo Fisher Scientific) and 9 (Cat. No. KHC3081, Thermo Fisher Scientific) were estimated using the ELISA’s instruction.

### Quantitative real-time reverse transcription polymerase chain reaction

Total RNA from the hearts of the experimental rats was isolated with the Trizol reagent as the manufacturer’s instructions (Invitrogen, United States of America). The first strand of cDNA was synthesized by reverse transcription (20 μL) with a random primer by using total 2 µg of RNA. After a denaturing step of 1 min at 95°C, several polymerase chain reaction (PCR) cycles resulted in the amplification of β-actin. Primer list is shown in Table 1.

**Table 1:** List of genes and primer sequences.

S. No	Gene	Primers	
		Forwarded	Reverse
1	TNF-α	GTAGCCCACGTCGTAGCAAA	CCCTTCTCCAGCTGGAAGAC
2	IL-6	CTTCCAGCCAGTTGCCTTCT	GAGAG CATTGGAAGTTGGGG-
3	IL-10	TGCCTTCAGTCAAGTGAAGACT	AAACTCATTCATGGCCTTGTA
4	IL-1β	CAGCAGCATCTCGACAAG AG	AAAGAAGGTGCTTGGGTCCT
5	Bax	CCTGAGCTGACCTTGGAGCA	GGTGGTTGCCCTTTTCTACT
6	Bcl-2	TGATAACCGGGAGATCGTGA	AAAGCACATCCAATAAAAAGC
7	Caspase-3	CTCGGTCTGGTACAGATGTCGATG	GGTTAACCCGGGTAAGAATGTGCA
8	Caspse-9	AGCCAGATGCTGTCCCATAC	ACCTGGGAAGGTGGAGTAGG
9	β-actin	CCAGATCATGTTTGAGACCTTCAA	GTGGTACGACCAGAGGCATACA

TNF-α: tumor necrosis factor-α; IL: interleukin; Bax: Bcl-2-associated X protein; Bcl-2: B-cell lymphoma 2. Source: Elaborated by the authors.

### Statistical analysis

For statistical analysis, GraphPad Prism software was utilized. Dunnett’s T-test was used after a one-way analysis of variance (ANOVA), and *p* < 0.05 was deemed statistically significant.

## Results

### Electrocardiography parameters

**Figure 2:**
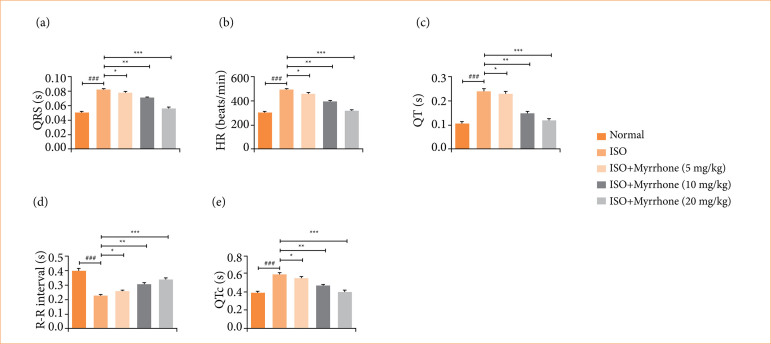
Effect of myrrhone on the electrocardiogram parameters of normal and isoproterenol (ISO)-induced myocardial injury group rats. **(a)** QRS, **(b)** heart rate (HR), **(c)** QT, **(d)** R-R and **(e)** QTc. Data are expressed as mean ± standard error of the mean (n = 6 for each group).

ECG parameters of myocardial injury rats induced by ISO are significantly different from those of control animals. In particular, the QRS complex, HR, QT interval, R-R intervals, and the corrected QT interval (QTc) were severely affected (Fig. 2a–2e). These alterations in ECG characteristics are a manifestation of the myocardial dysfunction and resultant electrical derangements of myocardial injury. ISO-induced myocardial injury model is commonly used to investigate the pathophysiology of myocardial injury and estimation the therapeutic interventions. Myrrhone treatment significantly (*p* < 0.001) reduced the ECG levels, indicating the possible cardioprotective activity of myrrhone. The modulation of ECG parameters by myrrhone demonstrates its competence to normalize the cardiac electrophysiological abnormalities induced by myocardial injury and possibly enhance the cardiac performance.

#*p* < 0.05 as compared to normal; *p < 0.05 as compared to ISO control analyzed by one-way analysis of variance followed by Dunnett’s T test. Source: Elaborated by the authors.

### Body weight, heart weight and heart weight/body weight ratio

**Figure 3:**
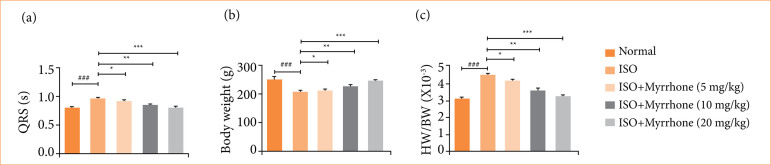
Effect of myrrhone on the body weight (BW), heart weight (HW) and HW/BW ratio of normal and isoproterenol (ISO)-induced myocardial injury group rats. **(a)** Heart weight, **(b)** body weight, and **(c)** HW/BW ratio. Data are expressed as mean ± standard error of the mean (n = 6 for each group).

Body weight (BW), heart weight (HW), and HW/BW ratio are indispensable physiological parameters for medical and biological studies. Body mass is a rough index of an individual’s size and general nutritional condition. HW correlates with the size and status of this essential organ. The HW/BW ratio is a derived index that corrects HW for differences in body size and facilitates comparability between smaller and larger individuals or groups. ISO-induced myocardial injury rats showed increased HW (Fig. 3a) and decreased BW (Fig. 3b). Myrrhone treatment significantly (*p* < 0.001) reduced HW and increased BW. In the ISO-induced myocardial injury group, rats exhibited an increased HW/BW ratio (Fig. 3c), which was significantly reduced (*p* < 0.001) by myrrhone treatment.

#*p* < 0.05 as compared to normal; *p < 0.05 as compared to ISO control analyzed by one-way analysis of variance followed by Dunnett’s T test. Source: Elaborated by the authors.

### Cardiac parameters

**Figure 4:**
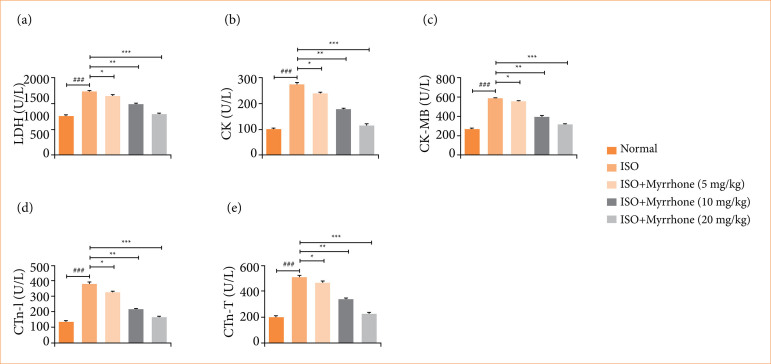
Effect of myrrhone on the biochemical parameters of normal and isoproterenol (ISO)-induced myocardial injury group rats. **(a)** lactate dehydrogenase (LDH), **(b)** creatine kinase (CK), **(c)** CK-MB, **(d)** cardiac troponin I (cTnI), and **(e)** cardiac troponin T (cTnT). Data are expressed as mean ± standard error of the mean (n = 6 for each group).

ISO group rats exhibited the boosted level of LDH (Fig. 4a), CK (Fig. 4b), CK-MB (Fig. 4c), CTnI (Fig. 4d), and CTnT (Fig. 4e). These are enzymes that usually enter the blood stream following damage or stress to cardiac muscle cells. Myrrhone treatment significantly (*p* < 0.001) repressed the level of cardiac parameters.

#*p* < 0.05 as compared to normal; *p < 0.05 as compared to ISO control analyzed by one-way analysis of variance followed by Dunnett’s T test. Source: Elaborated by the authors.

### Electrolytes

**Figure 5:**
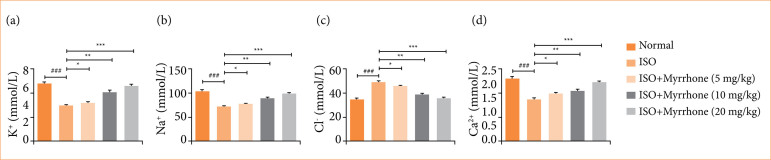
Effect of myrrhone on the electrolyte levels of normal and isoproterenol (ISO)-induced myocardial injury group rats. **(a)** K^+^, **(b)** Na^+^, **(c)** Cl^-^, and **(d)** Ca^2+^. Data are expressed as mean ± standard error of the mean (n = 6 for each group).

Electrolyte imbalance is a well-recognized consequence of myocardial injury, with significant implications for both cardiac function and overall physiological homeostasis. Myocardial injury was induced in rats with ISO, and it was found that concentrations of K^+^, Na^+^, Cl^-^, and Ca^2+^ underwent remarkable changes during the progress of myocardial injury. ISO induced myocardial injury rats showed the altered level of K^+^ (Fig. 5a), Na^+^ (Fig. 5b), Cl^-^ (Fig. 5c), and Ca^2+^ (Fig. 5d). Myrrhone treatment significantly (*p* < 0.001) modulated the electrolyte level.

#*p* < 0.05 as compared to normal; *p < 0.05 as compared to ISO control analyzed by one-way analysis of variance followed by Dunnett’s T test. Source: Elaborated by the authors.

### Membrane-bound enzyme

**Figure 6:**
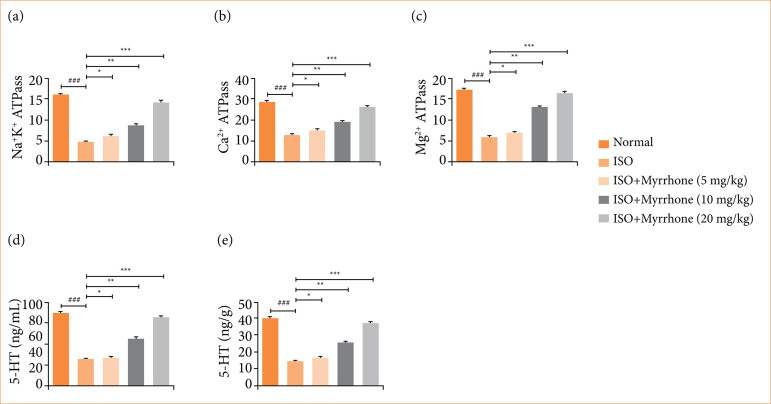
Effect of myrrhone on the membrane bound enzyme and 5-HT parameters of normal and isoproterenol (ISO)-induced myocardial injury group rats. **(a)** Na+K+ ATPase, **(b)** Mg2+ ATPase, **(c)** Ca2+ ATPase, **(d)** 5-HT in serum, and **(e)** 5-HT in tissue. Data are expressed as mean ± standard error of the mean (n = 6 for each group).

The membrane-bound enzymes play a crucial role in maintaining electrochemical gradients across cell membranes, regulating intracellular ion concentrations, and facilitating various physiological processes. The decrease in their activity suggests compromised cellular energy metabolism and impaired ion transport mechanisms, which are characteristic features of myocardial damage during myocardial injury. The level of Na^+^K^+^ ATPase (Fig. 6a), Mg^2+^ ATPase (Fig. 6b), and Ca^2+^ ATPase (Fig. 6c) reduced in the ISO-induced myocardial injury group rats and myrrhone treatment significantly (*p* < 0.001) boosted the level of membrane-bound enzymes. This restoration of enzyme activity suggests that myrrhone possesses cardioprotective properties, potentially through its ability to stabilize cell membranes, improve energy metabolism, and restore ion homeostasis.

#*p* < 0.05 as compared to normal; *p < 0.05 as compared to ISO control analyzed by one-way analysis of variance followed by Dunnett’s T test. Source: Elaborated by the authors.

### 5-hydroxytryptamine

The findings suggested that reduced ISO-induced myocardial injury in rats may be accompanied by reduced levels of 5-hydroxytryptamine (HT) in serum and tissue. Such decrease in 5-HT level may be involved in the pathogenesis of myocardial injury and could influence the cardiac function, platelet aggregation and vascular tone. ISO induced myocardial injury group rats showed the suppressed level of 5-HT in the serum (Fig. 6d) and tissue (Fig. 6e), and myrrhone treatment significantly (*p* < 0.001) boosted the level in serum and tissue. The substantial rise of 5-HT level induced by myrrhone treatment may have great implications for cardiac recovery and function post-myocardial injury and may contribute to improve cardiovascular outcomes via better regulation of heart rate, blood pressure, and platelet function.

### Hepatic parameters

**Figure 7:**
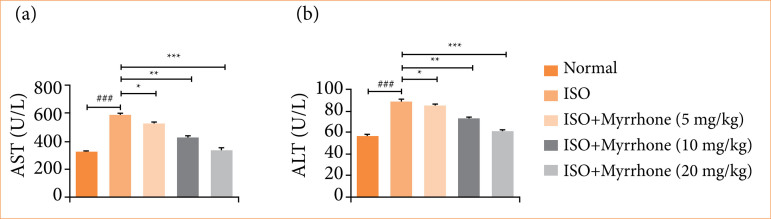
Effect of myrrhone on the level of hepatic parameters of normal and isoproterenol (ISO)-induced myocardial injury group rats. **(a)** aspartate aminotransferase (AST), and **(b)** alanine transaminase (ALT) in serum. Data are expressed as mean ± standard error of the mean (n = 6 for each group).

ISO-induced myocardial injury group rats showed the enhanced level of hepatic parameters like aspartate aminotransferase (AST) (Fig. 7a), and alanine transaminase (ALT) (Fig. 7b). These enzymes are a common indicator of liver function or liver injury, and their elevation in the blood is indicative of hepatocellular injury or liver damage. The increase in these indices in the ISO-induced myocardial injury group may reflect secondary effects of myocardial injury on liver function, potentially associated with hemodynamic changes or a systemic inflammatory response resulting from cardiac damage in the rat model. Myrrhone ability to decrease the levels of AST and ALT may indicate that it might attenuate liver function and integrity during cardiac stress, perhaps through anti-inflammatory or antioxidant potentials.

#*p* < 0.05 as compared to normal; *p < 0.05 as compared to ISO control analyzed by one-way analysis of variance followed by Dunnett’s T test. Source: Elaborated by the authors.

### Lipid parameters

**Figure 8:**
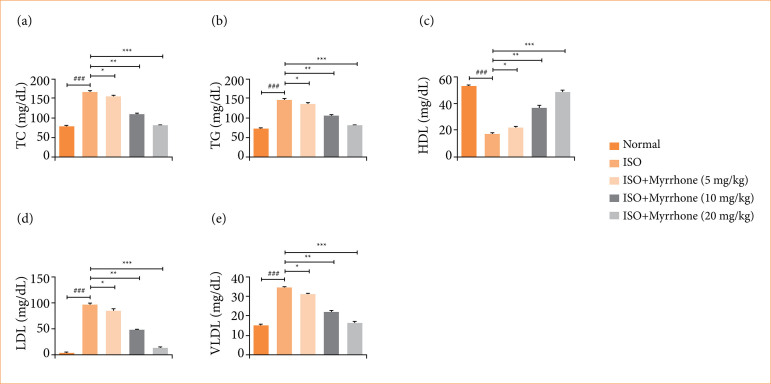
Effect of myrrhone on the lipid parameters of normal and isoproterenol (ISO)-induced myocardial injury group rats. **(a)** Total cholesterol (TC), **(b)** triglycerides (TG), **(c)** high-density lipoprotein (HDL), **(d)** low-density lipoprotein (LDL), and **(e)** very low-density lipoprotein (VLDL). Data are expressed as mean ± standard error of the mean (n = 6 for each group).

ISO group rats exhibited the altered level of lipid parameters like TC (Fig. 8a), TG (Fig. 8b), high-density lipoprotein (HDL) (Fig. 8c), low-density lipoprotein (LDL) (Fig. 8d), and very low-density lipoprotein (VLDL) (Fig. 8e). Such alterations of the lipid profile reflect the disturbed lipid metabolism and may be linked to an elevated cardiovascular risk profile. Myrrhone treatment significantly (*p* < 0.001) altered the level of lipid parameters. Modulation of these lipid parameters by myrrhone may be useful in treatment of lipid-related disorders or as cardioprotective agent.

#*p* < 0.05 as compared to normal; *p < 0.05 as compared to ISO control analyzed by one-way analysis of variance followed by Dunnett’s T test. Source: Elaborated by the authors.

### Oxidative stress parameters

**Figure 9:**
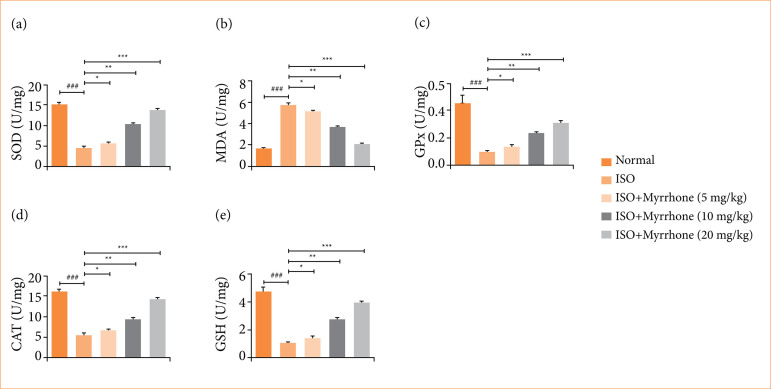
Effect of myrrhone on the antioxidant parameters of normal and isoproterenol (ISO)-induced myocardial injury group rats. **(a)** superoxide dismutase (SOD), **(b)** malondialdehyde (MDA), **(c)** glutathione peroxidase (GPx), **(d)** catalase (CAT), and **(e)** reduced glutathione (GSH). Data are expressed as mean ± standard error of the mean (n = 6 for each group).

ISO-induced myocardial injury group rats showed the altered levels of SOD (Fig. 9a), MDA (Fig. 9b), GPx (Fig. 9c), CAT (Fig. 9d), and GSH (Fig. 9e), and myrrhone treatment significantly (*p* < 0.001) modulated the level of oxidative stress parameters.

#*p* < 0.05 as compared to normal; *p < 0.05 as compared to ISO control analyzed by one-way analysis of variance followed by Dunnett’s T test. Source: Elaborated by the authors.

### Cytokines

**Figure 10:**
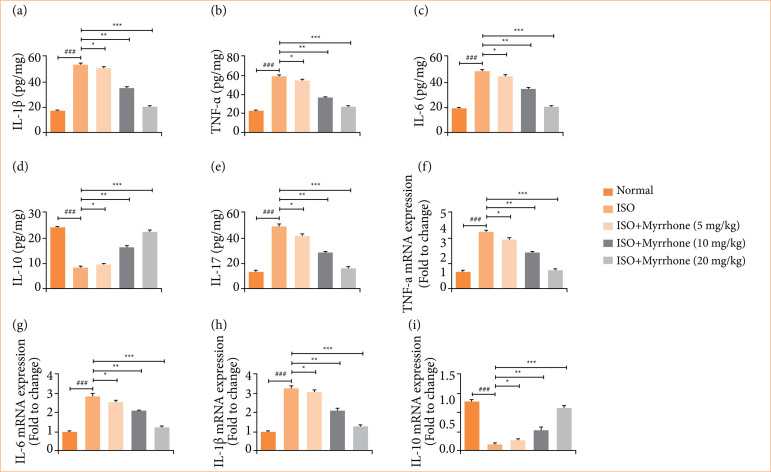
Effect of myrrhone on the inflammatory cytokines in tissue and inflammatory cytokines mRNA expression of normal and isoproterenol (ISO)-induced myocardial injury group rats. **(a)** Interleukin (IL)-1β, **(b)** tumor necrosis factor (TNF)-α, **(c)** IL-6, **(d)** IL-10, **(e)** IL-17, **(f)** TNF-α, **(g)** IL-6, **(h)** IL-1β and **(i)** IL-10. Data are expressed as mean ± standard error of the mean (n = 6 for each group).

ISO-induced myocardial injury group rats exhibited the altered level of cytokines like IL-1β (Fig. 10a), TNF-α (Fig. 10b), IL-6 (Fig. 10c), IL-10 (Fig. 10d), and IL-17 (Fig. 10e). Modifications of cytokine responses are consistent with those of the post-myocardial injury inflammatory reaction. The deregulation of these cytokines is involved in the pathophysiology of myocardial injury, leading to tissue injury, the remodeling of the heart and dysfunction. Myrrhone treatment significantly (*p* < 0.001) restored the level of inflammatory cytokines. This indicates that myrrhone has strong anti-inflammatory activity over inflammation in myocardial injury.

#*p* < 0.05 as compared to normal; *p < 0.05 as compared to ISO control analyzed by one-way analysis of variance followed by Dunnett’s T test. Source: Elaborated by the authors.

ISO-induced myocardial injury group rats showed altered mRNA expression of cytokines such as TNF-α (Fig. 10f), IL-6 (Fig. 10g), IL-1β (Fig. 10h), and IL-10 (Fig. 10i), and myrrhone treatment significantly (*p* < 0.001) modulated the mRNA expression.

### Inflammatory parameters

**Figure 11:**
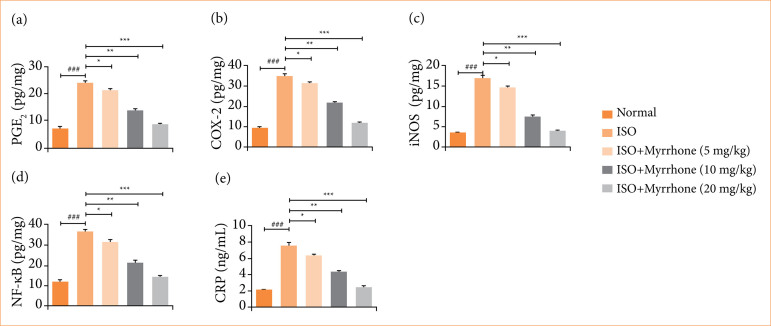
Effect of myrrhone on the inflammatory parameters of normal and isoproterenol (ISO)-induced myocardial injury group rats. **(a)** Prostaglandin (PGE2), **(b)** cyclooxygenase-2 (COX-2), **(c)** inducible nitric oxide synthetase (iNOS), **(d)** kappa-light-chain enhancer of activated B cells (NF-κB), and **(e)** C-reactive protein. Data are expressed as mean ± standard error of the mean (n = 6 for each group).

The myocardial injury group rats exhibited the increased levels of various inflammatory cytokines, which suggested a severe inflammatory response after suffering from myocardial injury. ISO-induced myocardial injury group rats showed the increased level of PGE2 (Fig. 11a), COX-2 (Fig. 11b), iNOS (Fig. 11c), NF-κB (Fig. 11d), and C-reactive protein (Fig. 11f). These inflammatory mediators are critical for the advancement of myocardial injury and subsequent repair following injury. Myrrhone treatment significantly (*p* < 0.001) suppressed the level of inflammatory parameters. The ability of myrrhone to reduce several inflammatory mediators at a time suggests that myrrhone could serve as a treatment for post-infarct inflammation and possibly overall cardiac outcomes.

### Apoptosis parameters

**Figure 12:**
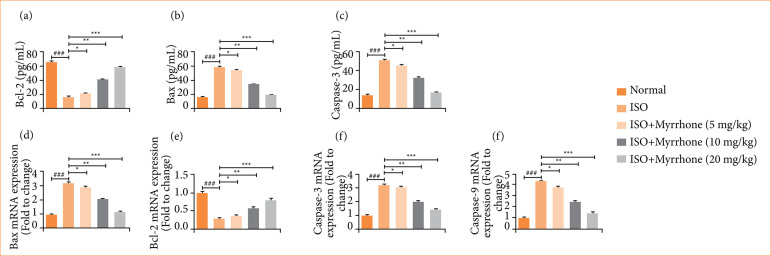
Effect of myrrhone on the apoptosis parameters and mRNA expression of normal and isoproterenol (ISO)-induced myocardial injury group rats. **(a)** B-cell lymphoma 2 protein (Bcl-2), **(b)** Bcl-2-associated X protein (Bax), **(c)** caspase-3, **(d)** Bcl-2 mRNA, **(e)** Bax mRNA, **(f)** caspase-3 mRNA, and **(g)** caspase-9 mRNA. Data are expressed as mean ± standard error of the mean (n = 6 for each group).

ISO-induced myocardial injury group rats demonstrated the altered level of Bcl-2 (Fig. 12a), Bax (Fig. 12b), and caspase-3 (Fig. 12c). They reflect a disturbance in the equilibrium between pro-apoptotic and anti-apoptotic factors, which may result in enhanced cell death in the myocardium. Myrrhone treatment significantly (*p* < 0.001) restored the level of apoptosis parameters. This normalization of apoptotic equilibrium may be responsible for diminution of death of cardiac-myocytes and normalization of myocardial function, suggesting that the myrrhone possesses potential therapeutic implications for myocardial injury and other related cardiac disorders.

ISO-induced myocardial injury group rats showed the altered mRNA expression of Bax mRNA expression (Fig. 12d), Bcl-2 mRNA expression (Fig. 12e), caspase-3 mRNA expression (Fig. 12f), and caspase-9 mRNA expression mRNA expression (Fig. 12g), and myrrhone treatment significantly (*p* < 0.001) modulated the mRNA expression.

#*p* < 0.05 as compared to normal; *p < 0.05 as compared to ISO control analyzed by one-way analysis of variance followed by Dunnett’s T test. Source: Elaborated by the authors.

#### MMP

**Figure 13:**
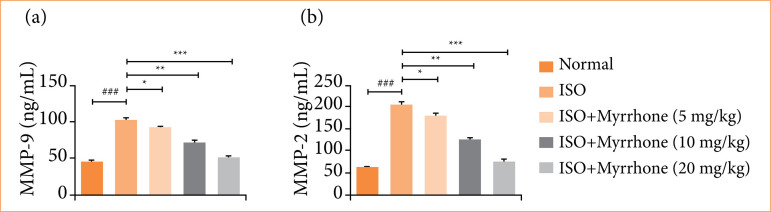
Effect of myrrhone on the MMP level of normal and isoproterenol (ISO)-induced myocardial injury group rats. **(a)** MMP-9, and **(b)** MMP-2. Data are expressed as mean ± standard error of the mean (n = 6 for each group).

ISO-induced myocardial injury group rats exhibited the boosted level of MMP-9 (Fig. 13a), and MMP-2 (Fig. 13b). This MMP upregulation is a typical constituent of the pathologic remodeling process after myocardial damage. The elevated MMP activity has a role in the disruption of structural proteins in the heart, resulting in the adverse cardiac remodeling, ventricular dilation, and heart failure. Myrrhone treatment significantly (*p* < 0.001) suppressed the MMP level.

## Discussion

Myocardial injury causes permanent damage to heart tissue and increases the mortality rate among affected individuals^24^,^25^. The pathogenesis of myocardial injury involves several processes, such as cardiac injury, oxidative stress, contractile dysfunction, calcium overload, and cell death through apoptosis, necrosis, or both4. The ISO-induced myocardial injury model is a well-established and non-invasive approach for reliably evaluating the cardioprotective potential of various therapeutic agents^4^,^26^.

Previous reports suggested that the secretion of 5-HT begins during the acute inflammation, cardiac ischemia, and tissue injury, primarily from platelets^27^. During these conditions, 5-HT secreted from the platelets4. Thrombus formation, platelet aggregation, coronary artery spasms, and smooth muscle cell contraction all are triggered by the secretion of additional 5-HT from the dense granules of platelets after it binds to 5-HT2A receptors^27^,^28^. The myocardial interstitium accumulates 5-HT, which contributes to cardiac cellular damage through various pathways, including 5-HT2A and 5-HT2B receptors, as well as 5-HT uptake transporters^4^,^28^.

A heart attack, also known as myocardial injury, occurs when blood flow to part of the heart is blocked long enough that part of the heart muscle is damaged or dies. Often the blockage is due to a buildup of fat, cholesterol, and other substances that creates a plaque in the coronary arteries^20^. When blood flow is disrupted, the affected heart tissue is deprived of oxygen and nutrients, leading to cell injury and death. This can cause significant damage to the heart muscle, impairing its ability to pump blood effectively^5^,^7^,^14^. When blood flow is restored, an inflammatory reaction and oxidative stress are initiated, which play a crucial role in damaging cells and tissues. During myocardial injury, various cardiac biomarkers such as CK-MB, LDH, cTnT, and cTnI are secreted into the blood^1^,^13^. Elevated levels of these biomarkers indicate myocardial necrosis (death of heart muscle cells) and cell membrane injury. These biomarkers are used to estimate, diagnose, and monitor the severity of myocardial injury. An abnormally high level of CK-MB during myocardial injury is considered a specific biomarker for assessing myocardial injury. CK-MB and CK levels significantly increase within 3–4 hours after myocardial injury^7^,^29^. LDH is an enzyme found in many tissues throughout the body, including the heart. CK-MB and LDH are also used as biomarkers for diagnose the myocardial injury. cTnI is a protein found in heart muscle and is highly specific for myocardial injury. Cardiac troponins are the preferred biomarkers for diagnosing acute myocardial injury^30^,^31^. Elevated levels indicate heart muscle damage and are used to assess the severity of the injury. cTnT, like cTnI, is a protein specific to the heart muscle and is a critical marker for myocardial injury. cTnT is used alongside cTnI to diagnose myocardial injury and evaluate the extent of heart muscle damage^7^,^30^. During myocardial injury, the heart muscle begins to sustain damage, leading to the secretion of these enzymes into the blood. The myrrhone treatment ameliorate the cardiac enzymes, which suggest the cardioprotective effect.

Na^+^, Cl^-^, K^+^, and Ca^2+^ are crucial electrolytes involved in the proper functioning of the heart. Their roles in myocardial injury are significant due to their influence on various physiological processes, including cellular signaling, muscle contraction and fluid balance^32^,^33^. Na^+^ is essential for generating action potentials in cardiac cells, facilitating electrical conduction, and maintaining osmotic balance. Chloride helps to maintain the electrical neutrality and osmotic balance within cells. It also participates in the regulation of cell volume and pH. During myocardial injury, sodium influx into cardiac cells increases the level of Cl^-^, leading to cellular swelling and potentially contributing to cell death. Increased intracellular sodium can also lead to calcium overload through the sodium-calcium exchanger, exacerbating injury^33^–^36^. Changes in Cl^-^ levels can influence the acid-base balance and contribute to arrhythmias during myocardial injury. Chloride shifts can affect cellular excitability and exacerbate damage in ischemic conditions. K^+^ is critical for repolarization of cardiac cells after a potential action. It helps maintain resting membrane potential and overall cardiac rhythm. During myocardial injury condition, potassium can leak out of damaged cells, leading to hyperkalemia in the extracellular space. This can cause arrhythmias and impair cardiac function. Additionally, hypokalemia can occur, further destabilizing cardiac electrical activity. Calcium is vital for excitation-contraction coupling in cardiac muscle cells. It triggers contraction by binding to troponin and facilitates the release of neurotransmitters. Calcium overload is a major factor in myocardial injury. Excessive calcium influx into cells can activate proteolytic enzymes, disrupt mitochondrial function, and lead to cell death. This calcium overload can result from ischemia-reperfusion injury, in which reintroduction of blood supply after ischemia leads to a surge in calcium influx^32^,^33^,^37^. Myrrhone treatment effectively restored the level of electrolyte levels towards the normal physiological ranges, which indicate that electrolytes (sodium, calcium, potassium, and chloride) are the indicative of the impaired cardiac function and systemic imbalance. Myrrhone treatment enhanced the level of electrolyte, suggesting the corrective effect on ISO-induced electrolyte disturbances.

ISO, through its sympathetic stimulation, can cause the dysregulation of lipid metabolism and increase the serum lipid levels, thereby contributing to a higher risk of coronary heart disease and myocardial injury^13^. It may stimulate the biosynthesis of lipids in the liver. An increase in acyl coenzyme A (acyl CoA) levels can lead to higher lipid synthesis. Elevated production of glycerol can contribute to triglyceride synthesis and accumulation^38^,^39^. Dysregulation of lipid metabolism is a significant factor in the development of myocardial injury. ISO is a synthetic catecholamine that mimics the effects of the sympathetic nervous system by stimulating beta-adrenergic receptors^7^. Its cardiovascular effects include increased heart rate, cardiac output, and myocardial oxygen consumption. Previous reports have shown that ISO administration leads to elevated levels of serum lipids, including LDL, TC, VLDL, and TG^13^,^38^. High levels of LDL and VLDL and low levels of HDL are associated with an increased risk of coronary heart disease and myocardial injury^13^. ISO administration induced the significant dyslipidemia, evidence via enhanced the serum levels of LDL, TC, TG, and VLDL and reduced the HDL. Myrrhone treatment considerably restore these alterations, restoring lipid profile balance. The lipid lowering effect of myrrhone treatment suggests its potential role to mitigate ISO-induced atherogenic risk and also contributing to its overall cardioprotective mechanism.

The overproduction of ROS, combined with depleted antioxidant defenses, leads to oxidative stress, triggering a series of pathological events that cause significant functional and structural damage to cardiomyocytes^26^,^40^. During myocardial injury, there is a massive production of ROS, particularly due to the autooxidation of ISO. These ROS can attack various molecules, with a particular affinity for polyunsaturated fatty acids in cell membranes. ROS target polyunsaturated fatty acids, leading to the formation of peroxyl radicals. These radicals initiate a chain reaction of lipid peroxidation, a key pathogenic event in myocardial necrosis. The resulting lipid hydroperoxides damage cardiac cell membranes and other cellular components^4^. The pathogenesis of myocardial injury involves a complex interplay of oxidative stress and lipid peroxidation. Excessive production of ROS leads to the peroxidation of membrane lipids, resulting in cellular damage and necrosis^1^. MDA serves as a critical biomarker for oxidative stress and lipid peroxidation in myocardial injury. Elevated MDA levels indicate increased lipid peroxidation and oxidative stress. The presence of high MDA levels in the ISO-treated group confirms the occurrence of oxidative stress in myocardial injury. GSH is a crucial non-enzymatic antioxidant that helps neutralize ROS. In myocardial injury, GSH levels drop significantly due to excessive ROS production. This depletion of GSH further exacerbates oxidative stress and cellular damage^1^,^41^,^42^. In the current study, the ISO administration considerably enhanced the MDA level and reduced the level of GPx, CAT, SOD, and GSH activities, suggesting the increased oxidative stress and myrrhone treatment significantly restored the levels of these parameters. The alteration in the level of antioxidant parameters suggest the potent free radical scavenging effect and antioxidant capacity. The antioxidant nature of myrrhone contribute to its cardioprotective potential against ISO induced myocardial injury in rats.

Myocardial injury is often accompanied by excessive generation of inflammatory cytokines, which exacerbates tissue damage^13^. IL-1β induces intense inflammatory reaction, which could induce tissue injury due to the enhancement of neutrophil invasion and other inflammatory factors. It reflects the degree of severity and highly correlates with the inflammatory level. Myocardial injury has been associated with raising levels of IL-1β, which complements cardiac damage via contributing to the inflammatory cascade. Several inflammatory reactions such as neutrophil development and mobilization are IL-6 dependent. Cardiopathies contribute to increased IL-6, its high concentration enhancing inflammatory and toxic effects, while suppressing catalase activity^13^,^43^. TNF-α is another key cytokine involved in inflammation. Its production is regulated by NF-κB, a transcription factor central to the inflammatory response. TNF-α exacerbates tissue damage and dysfunction in myocardial injury. Under normal conditions, NF-κB is kept inactive in the cytoplasm by inhibitory proteins called IκB. In response to inflammatory stimuli, IκB is phosphorylated and degraded, releasing NF-κB^44^–^46^. This allows NF-κB to translocate to the nucleus, in which it binds to promote sequences of target genes and induces the transcription of inflammatory cytokines such as IL-1β, IL-6, and TNF-α. Activation of the NF-κB pathway is a critical step in the inflammatory response during myocardial injury. It leads to the production of various inflammatory cytokines, amplifying the inflammatory response and contributing to tissue damage^13^,^44^. ISO considerably activated the NF-κB in the cardiac tissue, which further enhanced the production of inflammatory cytokines. Myrrhone treatment significantly ameliorated NF-κB activation, as evidenced via reduced the inflammatory response. The anti-inflammatory action directly contributes to enhanced the myocardial integrity and function.

Apoptosis is of great importance in the ISO-induced myocardial injury, which is mainly related to the imbalance of pro-apoptotic and anti-apoptotic signaling pathways. Increased expression of Bax and caspase-3 and decreased Bcl-2 expression induce cardiomyocyte apoptosis. The results of the present study showed that treatment with ISO up-regulated expression of Bax and caspase-3, and down-regulated expression of Bcl-2, indicating a promoted apoptosis. Reportedly, myrrhone treatment apparently revoked the pattern, resulting in inhibitions of Bax and caspase-3 and induction of Bcl-2 expression to restore the apoptotic equilibrium. These results emphasize the anti-apoptotic efficacy of myrrhone and indicate that its cardioprotective properties are, at least in part, through the maintenance of cardiomyocyte viability via the regulation of the Bax/Bcl-2/caspase-3 signaling pathway.

## Conclusion

The present study showed that myrrhone has a potent cardioprotective effect against ISO-induced myocardial injury in rats. Myrrhone treatment significantly ameliorated the ECG and physiological parameters, restored the antioxidant enzymes to their regular molecules, and ameliorated the cardiac and hepatic markers and the correcting the electrolytic imbalances. Moreover, it alleviated inflammatory and apoptotic reactions, as evidenced by a reduced expression of pro-inflammatory cytokines and pro-apoptotic markers, as well as increased anti-inflammatory and anti-apoptotic factors. Inhibiting NF-κB activation and regulating the Bax/Bcl-2/Caspase-3 signaling pathway are likely the molecular mechanisms responsible for myrrhone’s protective effects. These results indicate that myrrhone could be a potential drug for preventing or treating myocardial injury induced by oxidative stress, inflammation, and apoptosis.

## Data Availability

The data will be available upon request to the corresponding author.
